# The cognitive cost of age-related hearing loss

**DOI:** 10.1186/s12877-026-07476-w

**Published:** 2026-05-19

**Authors:** Mila Crnojević, Zoran Komazec, Slobodanka Lemajić-Komazec, Nikola Denda, Vojislava Bugarski Ignjatović

**Affiliations:** 1 Faculty of Medicine, University of Novi Sad, Novi Sad, Serbia; 2 University Clinical Center of Vojvodina, Clinic for Otorhinolaryngology and Head and Neck Surgery, Novi Sad, Serbia; 3 Faculty of Pharmacy, University Business Academy, Novi Sad, Serbia; 4 University Clinical Center of Vojvodina, Clinic for Eye Diseases, Novi Sad, Serbia; 5 University Clinical Center of Vojvodina, Clinic for Neurology, Novi Sad, Serbia

**Keywords:** Presbycusis, Age-related hearing loss, Cognitive decline, Quality of life

## Abstract

**Background:**

Age-related hearing loss (ARHL) or presbycusis is a bilateral sensorineural hearing impairment associated with aging of the structures of the inner ear and auditory pathways and is the most common cause of acquired hearing loss in older adults. Presbycusis is a disabling condition that affects communication and quality of life, and has been associated with poorer cognitive performance. This study aimed to examine the association between ARHL, cognitive screening performance, and hearing-related quality of life in older adults.

**Methods:**

This single-center prospective observational study with cross-sectional analysis included 60 participants aged 60 years and older: 40 patients with presbycusis and 20 control participants with normal hearing or mild hearing loss. Hearing threshold was examined using pure-tone audiometry and expressed as the Pure Tone Average (PTA). Cognitive function was screened using the Mini-Mental State Examination (MMSE) and Montreal Cognitive Assessment (MoCA), and quality of life was evaluated using the Hearing Handicap Inventory for the Elderly Screening Version (HHIE-S) questionnaire.

**Results:**

On cognitive screening, most patients with age-related hearing loss scored in the range suggestive of cognitive impairment, and 90% reported reduced hearing-related quality of life. Statistically significant correlations were observed between PTA and both MoCA and MMSE scores, suggesting that hearing threshold is strongly associated with cognitive status. Exploratory analyses also suggested an association between self-reported duration of hearing loss and cognitive screening results, although this finding should be interpreted cautiously.

**Conclusion:**

In this sample of older adults, worse hearing thresholds were associated with poorer performance on cognitive screening instruments and with lower hearing-related quality of life. These findings support further investigation of early hearing assessment and rehabilitation in older adults, and indicate the necessity of effective and timely auditory amplification, even in individuals with moderate hearing loss.

## Background

The global population is aging rapidly: by 2045, people aged over 60 will outnumber children under five worldwide, reaching nearly 2 billion [1]. This trend is seen across all regions, not just in the most developed ones, with population aging posing significant social and economic challenges, and highlighting the need for long-term planning and support systems [2–5].

Presbycusis, or age-related hearing loss (ARHL), is a bilateral sensorineural hearing impairment. It occurs as a result of aging of the structures of the inner ear and auditory pathways and is the most common cause of acquired hearing loss [6, 7]. Presbycusis typically becomes clinically apparent after the age of 60 and progressively worsens over time, although early auditory changes may begin as early as the age of forty. It leads to difficulties in various aspects of life, with the first symptom usually being difficulty in social communication [7]. Clinical presentation comprises progressive hearing loss, first registered in the high-frequency range, often associated with high-frequency tinnitus [8]. A specific characteristic of ARHL is impaired speech intelligibility, especially in conditions of increased background noise, since the consonants, which carry the intelligibility of speech, are articulated at higher frequencies [9]. ARHL is a disabling condition that affects all aspects of life. It impacts quality of life, social relationships, motor skills, psychological aspects, and the function and morphology of certain areas of the brain. Negative outcomes include increased risk of hospitalization, more frequent falls, inability to drive and general loss of independence [10–12].

With the population aging, the prevalence of ARHL increases, and it is estimated that one-third of the global population over the age of 65 suffers from disabling hearing loss [13]. However, currently, there is no effective treatment for ARHL. The mainstay of therapy is hearing aids, which do not restore hearing but only amplify it, while the auditory rehabilitation can also be applied with the goal of resocializing the patient [7].

Research over the past decade has shown there is a connection between presbycusis and cognitive decline, even with dementia, but it has not been well-studied. Although not fully understood, several mechanisms may explain this connection: self-isolation, as a consequence of impaired social communication, further leads to the development of various psychological disorders, such as depression, anxiety, reduced self-confidence and distrust of others, all of which are recognized risk factors for cognitive deterioration [14–17]. Further, hearing loss limits auditory input to the brain and reduces activity in regions responsible for hearing and speech, which, over time, may contribute to brain atrophy and accelerate cognitive decline [18–20]. Another possible mechanism concerns increased cognitive effort for speech comprehension of individuals with hearing impairment, thus leaving fewer cognitive reserves for other functions such as memory, attention, and planning [16, 17]. Finally, ARHL and cognitive decline may share vascular or neurodegenerative mechanisms, suggesting a common pathological substrate in the auditory pathway and brain, meaning that hearing loss could be an early indicator of overall brain aging [16, 17, 19].

Certainly, hearing loss is the third most disabling condition in older adults and is also one of the most significant risk factors for the development of cognitive decline and dementia [7, 16, 20]. This issue is particularly relevant in low- and middle-income countries such as Serbia, where state-funded provision of hearing amplification is often delayed until severe hearing loss has already developed, limiting its effectiveness in preventing cognitive decline [21]. Although numerous studies have examined the association between age-related hearing loss and cognitive decline, fewer have simultaneously evaluated hearing thresholds, duration of hearing loss, and cognitive performance while carefully accounting for age. Addressing these variables within the same analytical framework of this study may provide a more comprehensive understanding of the relationship between hearing status and cognitive function in older adults. Therefore, the aim of this study is to investigate the correlation between cognitive status and hearing loss as a reliable biomarker and a modifiable risk factor for the development of dementia.

## Materials and methods

### Study sample

This study was conducted as a prospective observational analytical cross-sectional study, involving a total of 60 participants of both sexes, aged over 60, divided into two groups: the study group consisting of 40 patients with presbycusis with moderate to severe hearing impairment, and the control group of 20 participants with normal hearing or with mild hearing loss. None of the patients with ARHL reported clinically significant tinnitus at the time of assessment. Fourteen participants in the ARHL group reported using hearing aids. However, all had been using the devices for less than three months at the time of assessment, which is generally considered insufficient to induce mesaurable cognitive or quality-of-life benefits. Therefore, hearing aid use was not analysed as a separate predictor.

The study was conducted at the Department of Audiology and Vestibulology of the Clinic for Otorhinolaryngology and Head and Neck Surgery at the University Clinical Center of Vojvodina (UCCV), Serbia, during 2024. The groups were comparable across all assessed sociodemographic variables.

*Inclusion Criteria* for the study sample were as follows:

- Individuals with presbycusis (only for the study group);
- Patients aged 60 years and over;
- At least four years of completed primary education were required to ensure basic literacy and adequate understanding of the cognitive tests.

*Exclusion Criteria* for study sample were defined as follows:

- History of significant occupational noise exposure;
- Severe visual impairment interfering with questionnaire completion;
- Known neurologic or psychiatric disorders, including previously diagnosed dementia or other cognitive disorders that could interfere with testing;
- Self-reported family history of dementia;
- Poorly controlled or unstable chronic diseases (e.g. uncontrolled diabetes or cardiovascular conditions) that could potentially influence cognitive performance.

### Methods

In this study, the following parameters were assessed in all participants: sociodemographic characteristics (gender and age) and clinical characteristics (hearing threshold, duration of hearing loss, cognitive status, and quality of life).

The assessment of hearing status was performed using audiometric testing. The results were expressed through Pure Tone Average (PTA), calculated as the mean value of hearing loss at the frequencies of 500 Hz, 1000 Hz, 2000 Hz and 4000 Hz, and expressed in on the better-hearing ear [22]. Hearing loss severity was classified according to pure-tone average (PTA) thresholds at speech frequencies. For the purposes of this study, hearing loss was grouped as mild (20–39 dB HL), moderate (40–59 dB HL), and severe (≥ 60 dB HL), broadly corresponding to commonly used audiological classification systems [23, 24] The control group included individuals with normal hearing or mild hearing loss, with a PTA cut-off value set at ≤ 35 dB HL at speech frequencies.

Cognitive status was evaluated using the Mini-Mental State Examination (MMSE^1^) and the Montreal Cognitive Assessment (MoCA). MMSE is a cognitive screening tool consisting of 11 tasks that assess five domains: orientation, perception, attention and calculation, recall, and language. The maximum score is 30, with scores of 24 or below indicating cognitive impairment [22, 25]. MoCA is a widely used and highly sensitive test for the early detection of mild cognitive impairment. It evaluates short-term memory, visuospatial abilities, executive functions, attention, concentration and working memory, language, and orientation to time and place. The maximum score is 30, with scores of 26 or higher considered normal [26–28]. To reduce the impact of hearing impairment on test administration, instructions were delivered face-to-face, slowly and clearly, repeated when necessary, and written support was provided where possible.

Finally, the quality of life in patients with ARLH was assessed using the Hearing Handicap Inventory for the Elderly Screening Version (HHIE-S). The questionnaire consists of 10 questions related to the social and emotional aspects of daily life, with possible answers of “yes” (4 points), “no” (0 points), and “sometimes” (2 points). Scores of 10–24 indicate mild to moderate disability, while scores of 26–40 indicate a significant degree of disability [29]. For the purposes of this study, HHIE-S questionnaire was translated into Serbian. The reliability of the translated version was assessed using Cronbach’s alpha method, with a value of 0.71, which is considered acceptable, confirming the reliability and consistency of the questionnaire [30].

In addition, the duration of hearing loss (DHL) was estimated primarily based on available medical history, including documentation of previous tonal audiometry examinations, when available. This information was supplemented by patient-reported onset and duration of subjective hearing difficulties, obtained during the clinical interview.

Occupational history was obtained during the clinical interview to identify possible lifetime exposure to excessive noise, and participants with a history of significant occupational noise exposure were excluded.

### Statistical analysis

The data obtained were analyzed and visualized using Microsoft Excel, while the statistical analyses were performed in Python with the SciPy library [31, 32]. Descriptive statistics were applied to summarize basic parameters (mean, standard deviation, minimum, and maximum). Pearson’s correlation coefficient was used to assess correlations between variables, and multiple linear regression was applied to evaluate the influence of predictors. Statistical significance was defined as *p* < 0.05.

### Ethics approval and consent to participate

All study procedures adhered to the Declaration of Helsinki [33], and the study protocol was approved by the Ethics Committee of UCCV (approval number 00–24 dated January 26th 2024, and approval number 00-282 dated July 31st 2025). Participation in the study was voluntary, anonymous and without financial compensation, and each participant provided informed consent before inclusion. A unique identification number was assigned to each participant, after which all data were entered into a secured database with restricted access available only to research team members. All analyses results were presented in an aggregated form, ensuring the complete anonymity of all participants.

## Results

### Study sample

Out of a total of 60 participants, 33 (55%) were women and 27 (45%) were men, with sociodemographic and clinical characteristics shown in Table 1.

**Table 1:** The structure of the study sample in terms of the age of participants, duration of the hearing loss (DHL), hearing status expressed through Pure Tone Average (PTA), cognitive status evaluated using the Mini-Mental State Examination (MMSE) and the Montreal Cognitive Assessment (MoCA), and the quality of life assessed using the Hearing Handicap Inventory for the Elderly Screening Version (HHIE-S)

Parameter	Group	Descriptive characteristics			
		min	max	Mean value	Stand. dev.
Age, [years]	Study	62.00	90	77.05	6.66
	Control	63.00	88	73.15	6.78
DHL, [years]	Study	0.25	60.00	12.86	16.01
	Control	n/a	n/a	n/a	n/a
PTA, [dBnHL]	Study	41.25	101.25	61.13	13.24
	Control	11.25	33.75	20.91	7.022
MoCA	Study	6.00	26.00	18.72	4.67
	Control	25.00	30.00	27.45	1.39
MMSE	Study	11.00	29.00	23.13	4.42
	Control	26.00	30.00	29.20	1.24
HHIE-S	Study	2.00	36.00	20.45	8.90
	Control	n/a	n/a	n/a	n/a

### Study group

The study group comprised 40 patients with presbycusis, including 21 women (52.5%) and 19 men (47.5%), with ages uniformly distributed between 62 and 90 years (mean age 77 years). Fourteen patients (35%) reported using hearing aids, however, duration and consistency of hearing aid use were not analysed separately in this study.

**Fig. 1:**
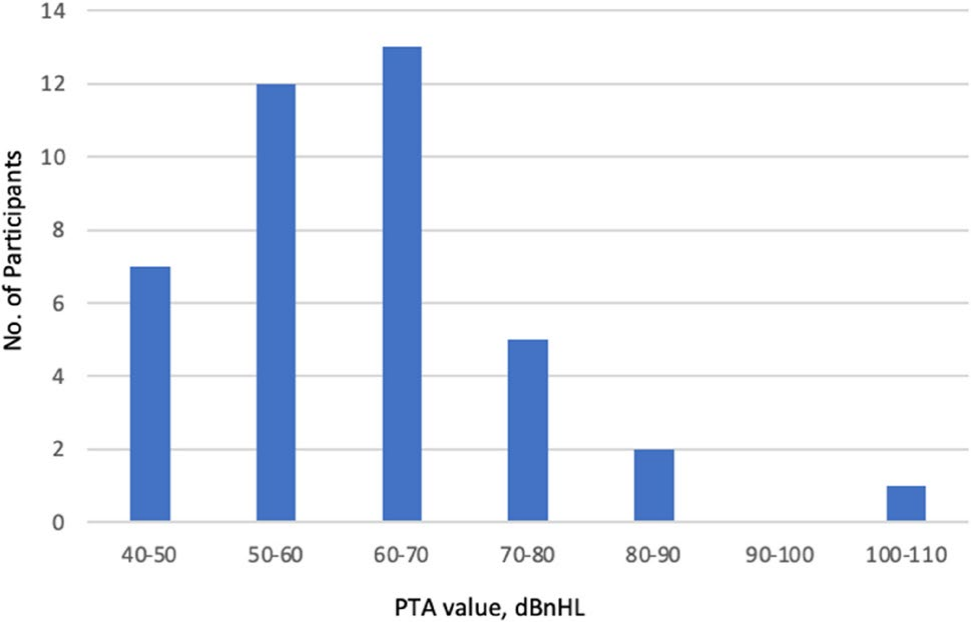
Distribution of patients by hearing impairment level

The results of audiometric tests showed that patients exhibited PTA values ranging from 41.25 to 101.25 dB nHL, Fig. 1, corresponding to 19 participants with moderate hearing loss, 20 with severe hearing loss, and 1 with total hearing loss.

**Fig. 2:**
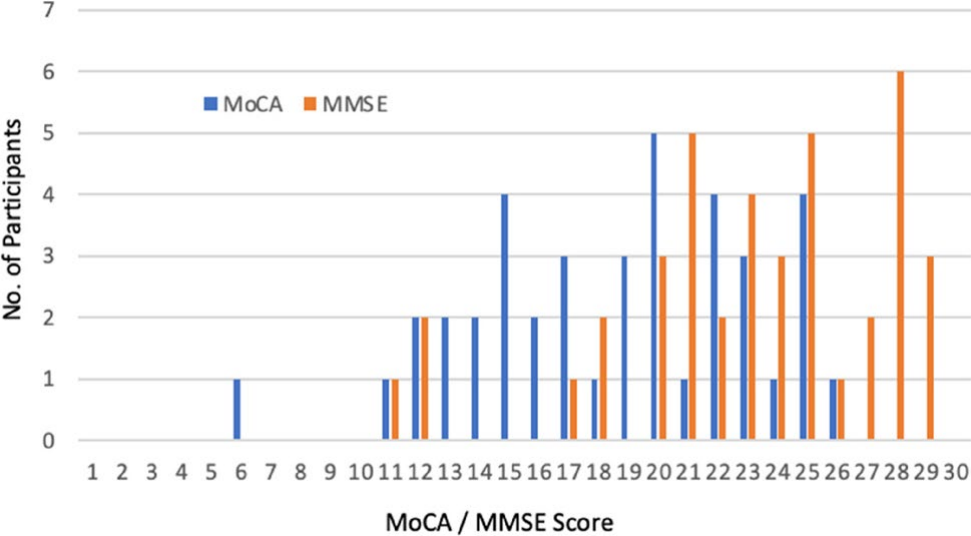
Cognitive status of patients with presbycusis assessed using MMSE and MoCA questionnaires

Cognitive status of the patients was assessed using the MoCA and MMSE questionnaires, Fig. 2. An indicative cut-off for cognitive impairment is generally considered to be a global score of ≤ 25 for the MoCA and ≤ 24 for the MMSE. In the study group, only 1 patient scored within the normal range on the MoCA, while 17 patients scored within the normal range on the MMSE. According to the screening results, 97.5% of patients scored in the range suggestive of cognitive impairment on the MoCA, while 57.5% showed scores suggestive of cognitive impairment on the MMSE.

The quality of life of the patients was analysed using the HHIE-S, and the following results were obtained: 4 (10%) patients denied significant subjective discomfort, indicating preserved quality of life, while 20 (50%) and 16 (40%) had moderate and severe impairment in quality of life, respectively. Overall, 90% of the patients with ARHL reported reduced quality of life.

### Control group

The control group consisted of 20 participants with normal hearing or mild hearing impairment, of whom 12 (60%) were women and 8 (40%) were men, with ages ranging from 63 to 88 years, and an average age of 73. Because these participants had preserved global cognitive screening scores and no self-reported hearing-related quality-of-life impairment, they were retained as a comparison group; however, the inclusion of individuals with mild hearing loss should be considered when interpreting between-group differences.

**Fig. 3:**
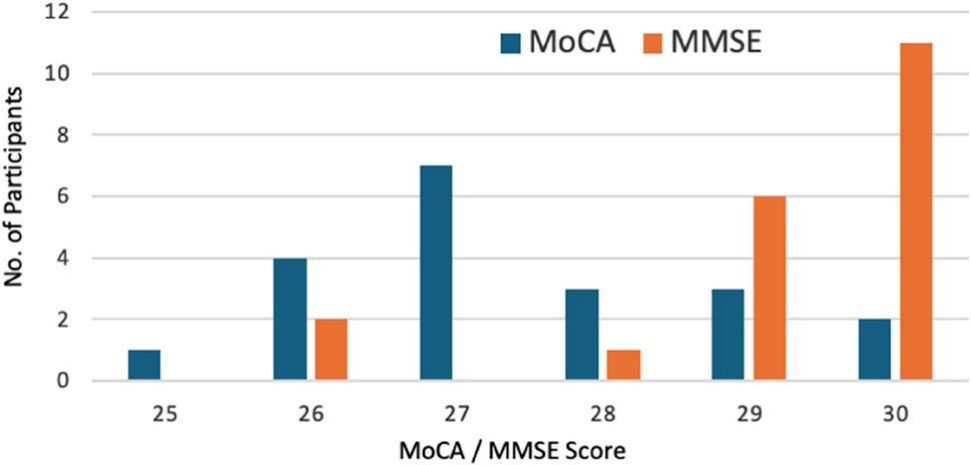
Cognitive status of participants with normal hearing or mild hearing impairment assessed using MMSE and MoCA questionnaires

All participants in the control group exhibited normal MoCA and MMSE global scores, except for one participant who obtained a MoCA score at the cut-off of 25, Fig. 3.

All participants were analyzed using the HHIE-S and denied significant subjective discomfort, meaning they had no impaired quality of life. Therefore, in the statistical analysis, the HHIE-S values for the control group participants were set to zero. Control-group participants denied clinically relevant hearing-related symptoms, therefore, DHL was coded as 0 for that group, which should be considered when interpreting DHL-related analyses.

### Sample analysis

**Fig. 4:**
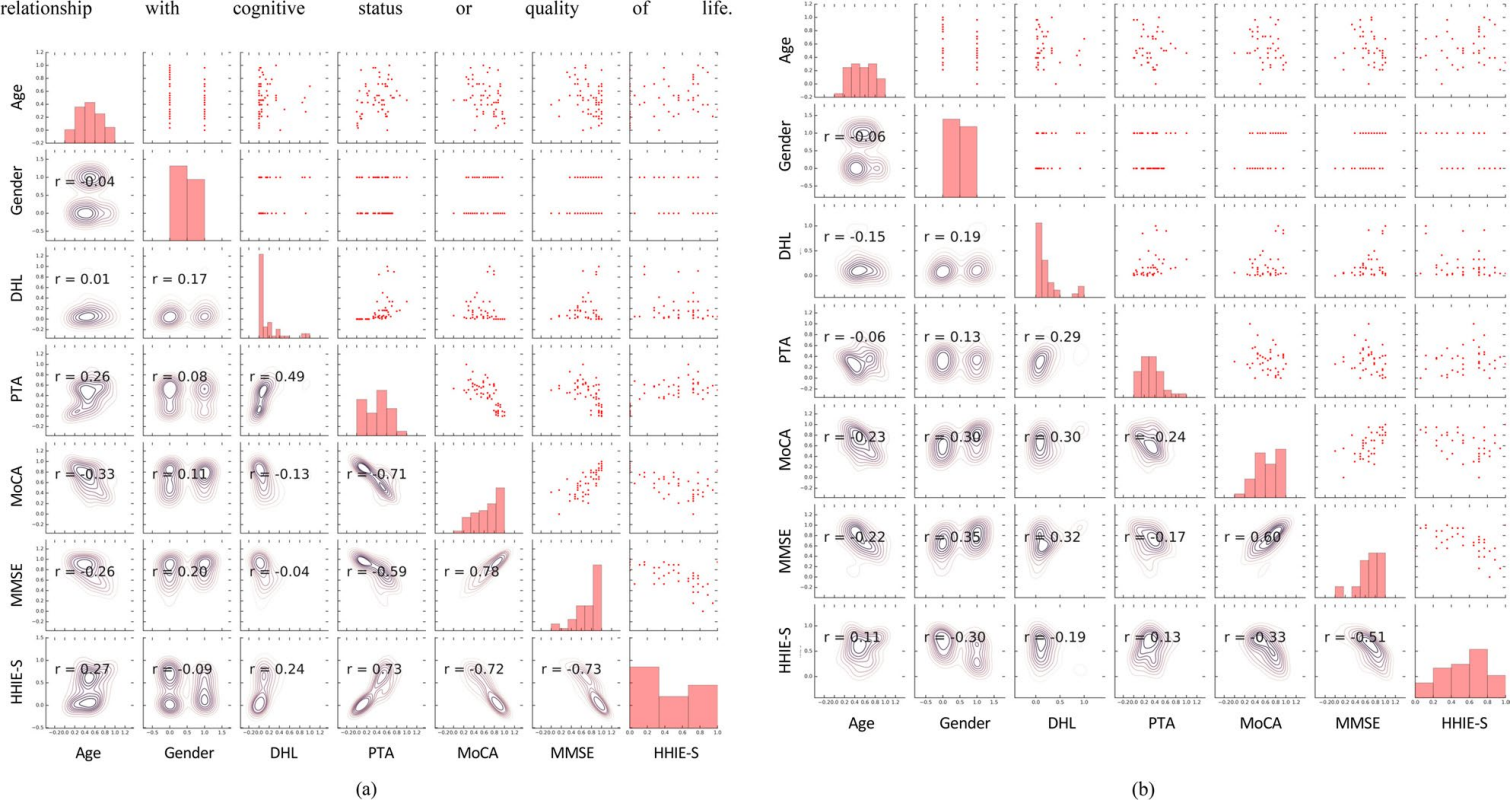
Pair plots illustrating relationships between variables for: (**a**) the entire sample and (**b**) the ARHL group. Histograms of individual variables are shown along the main diagonal. Scatter plots in the upper triangle display pairwise relationships between sociodemographic and clinical parameters, while the lower triangle shows the estimated two-dimensional density distributions. Pearson correlation coefficients (r) are provided to quantify the strength of pairwise associations

Pair plots were used as an exploratory visualization tool to examine correlations between the studied variables for the entire sample as well as for the ARHL group (Fig. 4). Since pair plots are descriptive rather than inferential, they are used only to guide, and not replace, the formal statistical analyses. Exploratory inspection suggests that PTA shows the clearest visual association with MoCA, MMSE, and HHIE-S scores, while weaker associations are observed for the duration of hearing loss (DHL) and age. Gender does not appear to show a clear relationship with cognitive status or quality of life.

### Results of statistical analysis

#### Results of linear correlation

The relationship between the duration of hearing loss and cognitive status was statistically examined for the entire study sample by determining the Pearson correlation coefficient between the results of MoCA, MMSE, and HHIE-S on one side, and the values of DHL and PTA on the other (Table 2).

**Table 2:** Pearson correlation coefficients for the study sample

Parameter 1	Parameter 2	Pearson corr. coeff.	*p*-value^*^
DHL	MoCA	-0.133	0.31
DHL	MMSE	-0.0398	0.76
DHL	HHIE-S	0.242	0.06
PTA	MoCA	-0.714	<<0.01
PTA	MMSE	-0.594	<<0.01
PTA	HHIE-S	0.729	<<0.01

^*^Significant correlations at *p* < 0.05

The analysis revealed a strong correlation between PTA on one side, and cognitive status and quality of life on the other, which aligns with the initial exploratory sample analysis In contrast, statistically significant correlations between the duration of presbycusis and MoCA/MMSE/HHIE-S were not observed in the entire sample. One important reason is that the control sample included individuals with mild hearing loss (PTA 11.25–33.50 dB), while DHL in the control group was coded as 0 since these participants denied clinically relevant hearing-related symptoms. This coding approach may have attenuated associations involving DHL and should be considered a methodological limitation of the study analysis (Table 2).

Therefore, the correlation analysis was repeated only in the study group, excluding the control group (Table 3). In this exploratory subgroup analysis, a statistically significant correlation was observed between DHL and MMSE, whereas the association between DHL and MoCA did not reach the conventional threshold for statistical significance, most likely due to the size of the sample. No clear association was observed between DHL and HHIE-S. Given the sample size and the partly self-reported nature of DHL, these findings should be interpreted cautiously.

**Table 3:** Pearson correlation coefficients for the study group

Parameter 1	Parameter 2	Pearson corr. coeff.	*p*-value^*^
DHL	MoCA	0.3	0.0581
DHL	MMSE	0.32	0.044
DHL	HHIE-S	-0,19	0.246

^*^Significant correlations at *p* < 0.05

#### Results of regression analysis

Linear regression (LR) was conducted to evaluate the influence of Age, Gender, PTA, and DHL on cognitive performance (MoCA and MMSE) and quality of life (HHIE-S), using the leave-one-out method, where model parameters were evaluated for all samples except one, which was then used for testing the model, formulated as follows:

$$\begin{aligned}\mathrm{m}\mathrm{o}\mathrm{d}\mathrm{e}\mathrm{l}\hspace{0.17em}&=\hspace{0.17em}{\upbeta\:}_{4}\:\bullet\:\:\mathrm{A}\mathrm{g}\mathrm{e}\hspace{0.17em}+\hspace{0.17em}{\upbeta\:}_{3}\:\bullet\:\:\mathrm{G}\mathrm{e}\mathrm{n}\mathrm{d}\mathrm{e}\mathrm{r}\hspace{0.17em}\\&+\hspace{0.17em}{\upbeta\:}_{2}\:\bullet\:\:\mathrm{D}\mathrm{H}\mathrm{L}\hspace{0.17em}+\hspace{0.17em}{\upbeta\:}_{1}\:\bullet\:\:\mathrm{P}\mathrm{T}\mathrm{A}\hspace{0.17em}+\hspace{0.17em}{\upbeta\:}_{0}\end{aligned}$$

Considering the sample size of 60 participants, the obtained coefficients of determination *R²* (Table 4) can be considered satisfactorily high.

**Table 4:** Linear regression (LR) analysis including all predictors (Age, Gender, PTA, DHL). Regression coefficients *β*_*i*_ indicate the strength and direction of each predictor’s effect (Age, Gender, DHL, and PTA, while 0 represents the model intercept), and the coefficient of determination *R²* indicates how well the regression model fits the data

	LR (MoCA)	LR (MMSE)	LR (HHIE-S)
R^2^	0.54	0.40	0.49
$\:{\beta\:}_{4}$	-2.66 ± 0.30	-1.23 ± 0.28	2.81 ± 0.63
$\:{\beta\:}_{3}$	1.50 ± 0.13	1.91 ± 0.11	-3.19 ± 0.30
$\:{\beta\:}_{2}$	5.95 ± 0.25	5.62 ± 0.26	-6.20 ± 0.83
$\:{\beta\:}_{1}$	-18.90 ± 0.30	-13.84 ± 0.3	38.16 ± 0.73
$\:{\beta\:}_{0}$	28.91 ± 0.14	29.68 ± 0.14	-0.83 ± 0.26

Since the quality of a statistical measure depends primarily on the nature of the predictors, the values of R² were also determined by using a reduced set of predictors, specifically by using only PTA and DHL individually, while the predictors Age and Gender were excluded from the linear regression model (Table 5). The values obtained using only PTA are very similar to those shown in Table 4, while no significant effect of DHL on MoCA, MMSE, or HHIE-S can be observed. In this sample, excluding Age and Gender did not materially change model performance, suggesting that PTA carried most of the predictive information for MoCA, MMSE, and HHIE-S scores in these models [34–36]. However, this should not be interpreted as evidence that age and gender are unimportant in general.

**Table 5:** Linear regression using only one predictor (PTA or DHL)

	LR (MoCA)	LR (MMSE)	LR (HHIE-S)
*R*^2^ using only PTA	0.48	0.32	0.50
R^2^ using only DHL	-0.05	-0.06	-0.02

Finally, the R² values were determined by using both PTA and DHL as predictors, while excluding Age and Gender from the linear regression model. (Table 6).

**Table 6:** Linear regression using PTA and DHL in combination

	LR (MoCA)	LR (MMSE)	LR (HHIE-S)
R^2^	0.54	0.39	0.50

Although Table 5 might suggest that DHL has limited predictive value, the R² values shown in Table 6 are practically identical to those obtained using all predictors (Table 4), and increased compared to the values obtained using only PTA (Table 5). From this, it can be concluded that the most significant influence on cognitive status (MoCA and MMSE) and quality of life (HHIE-S) is exerted by PTA and DHL together. Additionally, this result further confirms the importance of DHL as a factor shown in Table 3.

To further assess predictor stability and relative contribution, regression coefficients were evaluated across leave-one-out cross-validation (LOOCV) folds. To estimate their relative importance, predictors were ranked according to the absolute values of the stability indicator t, approximated as the ratio of the mean coefficient to its standard deviation. For MoCA, the ranking of predictors was: PTA (|t| = 63.36) > DHL (|t| = 23.29) > Gender (|t| = 11.66) > Age (|t| = 8.73). For MMSE, the ranking was: DHL (|t| = 46.34) > PTA (|t| = 21.58) > Gender (|t| = 16.67) > Age (|t| = 4.39). These results indicate that hearing-related variables show stronger associations with cognitive outcomes within the present model.

To account for uncertainty in coefficient estimates and to reduce the risk of overinterpretation, we additionally performed bootstrap resampling to obtain 95% confidence intervals for the regression coefficients. The results suggest that hearing-related variables (PTA and DHL) show consistent associations with cognitive performance, whereas the confidence intervals for Age and Gender include values close to zero, reflecting greater uncertainty related to the limited sample size and potential confounding. These findings indicate that hearing-related variables appear to contribute more strongly to cognitive outcomes within the present model, while the influence of Age and Gender is more modest and cannot be conclusively excluded.

## Discussion

The present study explored the association between presbycusis and cognitive screening performance in 60 older adults. Most patients with presbycusis scored below the conventional MoCA cut-off, whereas a smaller proportion scored below the MMSE cut-off, and 90% reported reduced hearing-related quality of life. The analysis showed statistically significant correlations between PTA and MoCA, MMSE, and HHIE-S values, respectively, as well as between the duration of presbycusis and cognitive status measured by MMSE. The most significant factor associated with cognitive decline in individuals with presbycusis was found to be the hearing threshold. Associations involving self-reported DHL were less consistent and should be interpreted cautiously, particularly because this variable depended largely on patient history and was operationalized differently in the control group.

These findings can also be interpreted in the broader context of the well-established relationship between age-related hearing loss and cognitive decline in older adults. Presbycusis has increasingly been recognized not only as a sensory impairment but also as a potential contributor to changes in cognitive functioning. Several neurobiological mechanisms have been proposed to explain this association, including increased cognitive load during auditory processing, reduced auditory input leading to cortical reorganization and sensory deprivation, as well as shared neurodegenerative and vascular pathways affecting both auditory and cognitive systems. In addition, hearing loss may indirectly influence cognitive health by promoting social withdrawal, reduced communication, and decreased cognitive stimulation. Despite growing interest in this relationship, important gaps remain in understanding how the severity and duration of hearing loss relate to cognitive performance and how these factors interact with aging and other health-related variables. In this context, examining hearing thresholds, duration of hearing loss, and cognitive screening outcomes within the same analytical framework may provide additional insight into the potential role of presbycusis as a modifiable risk factor for cognitive decline and dementia.

Our findings are broadly in line with other studies. A recent study from China reported an independent association between hearing loss and cognitive impairment, emphasizing depression as an additional factor [37]. Jiang et al. (2024) further supported a causal link through Mendelian randomization, focusing on specific subtypes of both hearing loss and dementia [38]. While their study provided genetic-level insight, our results add clinical data showing an association between worse hearing thresholds and lower cognitive screening scores.

Large cohort studies also provide evidence supporting this link. The Health ABC study from 2017 found an increased risk of dementia among individuals with moderate to severe hearing loss [39]. Similarly, the Health and Retirement Study (2021) showed a significantly increased risk of cognitive decline in older adults with moderate to severe hearing loss, further emphasizing the importance of treating hearing loss as a modifiable risk factor for dementia [40]. These results are consistent with our findings that hearing threshold is the most stable predictor of cognitive screening performance in our models. In contrast, the Busselton Baby Boomer study (2016) did not identify hearing loss as a predictor of cognitive decline, likely due to the younger age of participants and a very small number of individuals with severe hearing loss [41].

Other clinical studies also reported findings similar to ours. A 2015 study including 301 older adults showed that sensory deficits (hearing or vision) were associated with worse MoCA scores and greater risk of cognitive decline [42]. The studies by Lin et al. (2013, 2011) demonstrated that cognitive decline increases linearly with the degree of hearing loss [43, 44], while another study from the US found that hearing loss above 25 dB equates to approximately seven years of cognitive aging [45]. In our study, the association between hearing loss and cognitive screening performance was evident, but the small sample and limited characterization of hearing aid use precluded more detailed interpretation.

Evidence from Japan also supports this broader association. Sugawara et al. (2011) reported a clear link between hearing loss, cognitive decline, and poor quality of life, with additional influence of education, social functioning, and health status [46]. A study by Lim and Loo (2018) was very similar to ours and also associated presbycusis with worse MoCA and MMSE scores, while emphasizing the need to adapt cognitive tests for patients with impaired hearing [47]. Tong et al. (2022) confirmed the predictive value of PTA for MoCA and MMSE scores, especially in the right ear, though they did not include duration of hearing loss in their analysis [48].

Finally, interventional studies provide important insight into possible preventive strategies. The ACHIEVE trial (2023) showed that treating hearing loss with hearing aids and regular audiology follow-up significantly slowed cognitive decline in a subgroup of participants at higher risk [49]. A JAMA Neurology study (2023) confirmed the protective effect of consistent hearing aid use [50]. Similarly, a longitudinal study from the US confirmed that regular hearing aid use was associated with better cognitive outcomes over time [51]. Although our study was not designed to test treatment effects, these data support the clinical relevance of early identification and rehabilitation of hearing loss.

Overall, our results are consistent with a broader body of literature suggesting an association between presbycusis and poorer cognitive screening performance. In our analyses, PTA showed the most consistent relationship with MoCA, MMSE, and HHIE-S, whereas the role of DHL was less stable across models. Because both MMSE and MoCA include hearing-dependent components, lower scores in participants with ARHL may partly reflect test-administration constraints rather than cognitive status alone. Given the small, single-center, and age-imbalanced sample, these findings should be interpreted as preliminary. Future research should include larger and more strictly characterized control groups, long-term follow-up, cognitive screening tools adapted for hearing-impaired individuals, and a more detailed assessment of education, hearing-aid exposure, and other potential confounders.

The limitations of the study are as follows. This is a single-center study with a relatively small sample size, which may limit the generalizability of the findings and reduce the statistical power to detect more subtle associations between variables. In addition, the control group included individuals with mild hearing loss, which may have reduced the contrast between groups. The study groups were also not age-matched; therefore, although age was included as a covariate in the regression models, residual confounding related to age cannot be completely excluded.

Information on major medical conditions was collected during the clinical interview, and individuals with unstable or uncontrolled chronic diseases were excluded. However, vascular and lifestyle-related factors such as hypertension, diabetes, smoking, physical activity, depressive symptoms, and educational level were not systematically quantified and therefore could not be fully controlled for in the analysis. Family history of age-related hearing loss and lifetime noise exposure (apart from singnificant that were excluded from the study) were also not systematically assessed and may represent additional factors influencing hearing status.

In cases were medical history and previous tonal audiometry examinations were unavailable, the estimation of DHL relied on patient recall, which may introduce recall bias.

Furthermore, cognitive status was assessed using the MoCA and MMSE, which are screening tools rather than diagnostic instruments. The MoCA was not specifically adapted for individuals with hearing impairment (e.g., MoCA-HI). Although particular care was taken during test administration to minimize the potential influence of hearing loss - by providing clear and slow verbal instructions, written explanations when possible, and confirming participants’ understanding of each task - the potential impact of hearing difficulties on test performance cannot be completely excluded.

Finally, although participants were recruited prospectively, the present analysis is cross-sectional in nature, which limits the ability to infer causal relationships between hearing loss and cognitive decline. Accordingly, the findings should be interpreted as exploratory and hypothesis-generating.

## Conclusion

Presbycusis is a debilitating condition that impacts all aspects of life, and its prevalence increases with the aging population, thus making it a highly relevant research topic. It represents a significant disability for older adults and is one of the most important biomarkers and risk factors for the development of dementia.

The results of this exploratory single-center study, point to a significant association between age-related hearing loss (presbycusis) and cognitive decline. Age-related hearing loss was associated with poorer performance on cognitive screening instruments and with reduced hearing-related quality of life.

PTA showed the most consistent association with MoCA, MMSE, and HHIE-S, whereas findings related to duration of hearing loss were more exploratory. Although these results should not be interpreted as evidence of causality or of a treatment effect, they support further study of early hearing assessment and rehabilitation in older adults, and suggest the need for effective and timely hearing amplification, even for individuals with moderate hearing loss.

## Data Availability

All data generated or analyzed during this study are included in this published article [and its supplementary information files].

## Abbreviations

ARHL: Age-related hearing loss
PTA: Pure Tone Average
MMSE: Mini-Mental State Examination
MoCA: Montreal Cognitive Assessment
HHIE-S: Hearing Handicap Inventory for the Elderly Screening Version
UCCV: University Clinical Center of Vojvodina

## Footnotes

1
*An unauthorized version of the Serbian MMSE was used by the study team without permission*,* however this has now been rectified with PAR. The MMSE is a copyrighted instrument and may not be used or reproduced in whole or in part*,* in any form or language*,* or by any means without written permission of PAR (* www.parinc.com*).*
